# Hegemony to humanity: reimagining masculinity through Sam Miller’s adaptation of *A Gentleman in Moscow*

**DOI:** 10.3389/fsoc.2026.1816505

**Published:** 2026-05-26

**Authors:** A. Jerrin Steve, R. Preetha

**Affiliations:** Department of English, School of Social Science and Language, VIT Chennai, Chennai, India

**Keywords:** affective masculinity, authoritative, care, emotion, ethical masculinity, film studies, hegemonic masculinity, masculinities

## Abstract

The study examines the series *A Gentleman in Moscow* (2024), directed by Sam Miller, as a reconstruction of masculinity. This study focuses on tracing the transformation of the protagonist Count Alexander Rostov from a dominant form of masculinity, called hegemonic masculinity, to an ethical, affective, and relational responsibility. Rather than directing the focal point toward the fall of men, the study draws the decline of masculine authority from its aristocratic composure. As the events take place during the post-revolutionary Russian era, the study analyzes Rostov’s confinement within a closed enclosure as an inward moral recalibration. Rostov’s relationships with Nina as an instructor, with Anna as a partner, and with Sofia as a father present a progressive arc of masculinity from the initial stage of emotional literacy to romantic intimacy and ethical maturity. The research uses qualitative textual and visual analysis and synthesizes the frameworks of RW Connell’s hegemonic masculinity, habitus, and caring masculinity. The study also focuses on cinematic grammar, such as framing, lighting, deliberate pacing, and gestures, to perform masculinity’s ethical evolution. The traditional Russian context of endurance and restraint makes the adaptation read as culturally specific. Despite that, it resonates globally with post-patriarchal masculinity. The investigation concludes that the “A Gentleman in Moscow” series offers a model for ethical masculinity grounded not in power but in care.

## 1 Introduction

Over the past few decades, there has been a shift in the discourse on masculinity from a dominant to an affective one. Throughout human history, societies from different cultural backgrounds have always defined men by what they control rather than by what they feel. Masculinity is no longer understood as a stable or universal design but is studied in terms of historical performance with factors such as power, emotion, and circumstance. Observing the role of traditional masculinity, Connell (2005) studies men as emotionally restricted, dominant, and authoritative. An effectively built structure that encapsulates manhood through mastery, controlling his emotions and his environment. Thus far, this idea remains as a cultural inheritance rather than a biological truth. Connell and Messerschmidt (2005) further reformulates tradition as a hierarchy that constantly alters and reproduces itself through contrast against women and other subordinated masculinities. As Hearn (2014) argues, material and discursive forces inherently shape men’s practice of cultural expectations such as strength, control, and independence. These forces create a strain in which strength, autonomy, and assertiveness are necessary for being a man. Only when social expectations take a distorted turn from their traditional trajectory will the meaning of being a man begin to shift from hierarchy to humanity. This directional shift will mark one of the most subtle transformations in the modern understanding of masculinity.

This transformation of masculinity finds its most distinct re-examination in Miller’s (2024) adaptation of *A Gentleman in Moscow*. In this series, the main protagonist is stripped of his privilege and eventually redefines his identity via empathy. The adaptation dates back to the source of the 2016 novel, *A Gentleman in Moscow* by Amor Towles. The novel follows Count Alexander Rostov, a Russian aristocrat, who is imprisoned for life under house arrest in the Metrepol Hotel just after the Russian Revolution. He is confined to a single building with no possible way to contact the outside world. This collapses Rostov’s life outwardly through his confinement but expands inwardly, leading to self-discovery and moral rebirth. The very wall that limited his movement has opened a space for the awakening of his emotions. Thus, by breaking the stereotype of the gentleman as a holder of social hierarchy, he becomes an example of ethical reorientation. Through this confinement, Miller’s adaptation raises a profound question: What is masculinity without authority? The answer that the series provides as it progresses through each episode.

Such aesthetic reimagination is consistent with Svelstad’s (2024) idea of *affective adaptation*. He exemplifies that contemporary retellings of canonical masculinities will remap the emotional texture attached to them. In *A Gentleman in Moscow*’s series adaptation, this principle of *affective adaptation* is enumerated in a most effective sense. Instead of a simple narration of Towles’ novel, it restores its affective nucleus by transforming literary introspection into visual intimacy. The series instigates the cultural work that the theory describes as the conversion of dominance into care. For Russian masculinity, this transformation from “masculine heritage’ which was built on service, silence, and sacrifice, to reconciliation occurs through Rostov’s quiet acts of kindness and care”. It also articulates a masculinity that can survive historical upheaval by transforming inwardly rather than striking out.

The book *A Gentleman in Moscow* (Towles, 2016) establishes a new definition of what it means to be a “gentleman.” A term once used as a social distinction has evolved into a moral symbol that people now understand through their inner values rather than their ability to wield authority. The adaptation created by Sam Miller serves as a component that scholars can use to analyze masculinity and its associated emotional ethics. A movement pioneered by Connell’s sociology and Levant’s psychology, later followed by Hanlon’s ethics, and along with Svelstad’s framework, which bridges the sociological and philosophical theories of masculinity with cinematic grammar such as framing, lighting, pacing, and gesture. Thus, stating that they do not merely act as a decorative agent or visual aesthetics but as the means to study and analyze the masculine identity as the series builds.

## 2 Literature review

### 2.1 Masculinity as a structured practice

The cultural construction of masculinity has developed far beyond the basic understanding of manhood, while prevailing in the long struggle of rationalizing male dominance without naturalizing it. Being the foundation and pillar of masculinity studies, Connell’s (2005) hegemonic masculinity theory focuses on the social systems that enable patriarchal authority to persist throughout history. According to her, hegemonic masculinity is achieved through cultural performance and social approval, which reinforces the dominance of men and their mastery over other masculinities that are subordinate to hegemony. She also illustrates that men inherit stoicism through social learning, which creates the false belief that they lack a natural capacity for emotional empathy and intimacy. The further emphasis on “masculinities are configurations of practice structured by gender relations” (Connell, p. 44) provides a framework for understanding men and how they use their emotional control to conceal their social privileges. This masculinity reveals the paradox at the heart of “hegemony.”

Nevertheless, later theorists such as Hearn (2014) argue that Connell’s perspective can be overly structural. It encapsulates the framework of power, but underplays its ‘discursive and affective’ construction. Hearn’s notion of “material–discursive” states that only through the interaction between bodies and language (what is enacted and what is said about it) emerges masculinity as a whole. This argument opens the way for further discussion about “power” as something that is performed into existence through habitual acts and gestures. By combining Connell’s structural model with Hearn’s material-discursive approach, masculinity becomes a relational process rather than a fixed identity.

Bourdieu’s (2001) *Masculine Domination* offers a complementary perspective on gender from a sociological standpoint. He argues that masculine norms are not simply declared but absorbed and become naturalized through the concept of *habitus*. He states that external hierarchies are incorporated into bodily dispositions through actions such as moving, speaking, and perceiving the world, which may appear natural but are socially produced. What appears as natural masculine components are, in Bourdieu’s account, the body’s way of remembering its social history. Although this makes *habitus* a powerful tool for analyzing and understanding how deeply rooted masculine norms are, it reveals a limitation within the framework, which struggles to explain how these norms are genuinely transformed.

Butler’s (2004) work on *gender performativity* takes up precisely this question, through a philosophical distinction. According to Butler, gender is not something one has or expresses, but rather the repeated stylization of the body in relation to the social norms. Performance implies a subject who exists prior to the act and consciously steps into a role. Performativity, in contrast to performance, brings out how the subject itself comes into being through the act of repetition. A ritual that causes the illusion of essence rather than insisting on the innate truths. Through her work *Bodies That Matter* (Butler, 1993), she establishes the framework that every repetition (signifier) carries within it the slight possibility of deviation. “If the cultural construction of sexuality compels a repetition of that signifier, there is nevertheless in the very force of repetition, understood as resignification” (Butler, p. 89). Through this resignification, dominant meanings can be gradually reworked. Masculinity thus falls under performativity instead of a fixed structure, one that can be redefined and reconstructed through the repetition and reconfiguration of cultural and social acts.

Levant and Wong (2017), in *The Psychology of Men and Masculinities*, enumerates the psychological costs of men’s transformation. In which he states that achievement, control, and emotional restraint are considered the framework of manhood. For almost every man, what used to be called “strength” was often a cover for a fear of vulnerability, and what was labeled as “control” often concealed the suppression of emotion. Levant refers to this state of masculine façade as *normative male alexithymia*, the culturally acquired inability to perceive or express one’s feelings (Levant, p. 24). This psychological dimension of hegemonic masculinity is a crucial layer of what Rostov must work through across the series.

In contrast to hegemonic masculinity, scholars such as Hartung et al. (2024) discuss the ethical reorientation as the emergence of *affective* or *care masculinities*: models of manhood grounded in emotional intelligence and relational responsibility. Hanlon (2022) positions affective equality as a normative design with care at the center of equality, and where care ought to be recognized as a relational social practice rather than a private sentiment. Elliott (2016) explains that “caring masculinities are masculine identities that reject domination and its associated traits and embrace values of care such as positive emotion, interdependence, and relationality” (Elliot, p.241).

The theoretical framework is grounded in the work of Connell, Bourdieu, Butler, Hanlon, and Elliot. is linked to the specific medium of television adaptation through Svelstad’s (2024) concept of affective adaptation. He argues that contemporary retellings of canonical masculine narratives remap the emotional texture attached to the inherited masculine ideals. He further adds that the structure of dominance is directed to structures of care through the affective resources available to the medium.

### 2.2 Russian and post-soviet contexts of masculinity

Masculinity in Russia, compared to other national contexts, is rooted in tradition. It is more of a performance historically grounded in the ideals of nationality, religion, and politics. Russian masculinity, along with femininities, was revolutionized and reconstructed repeatedly through imperial, Soviet, and post-Soviet ideologies. From the aristocratic etiquette of the Tsarist court to the stoic collectivism of the Soviet worker, Russian manhood has always borne the weight of service and control. The following review combines key contributions that highlight how masculinity in Russia has been disciplined, moralized, and emotionally constrained across historical periods. Gorsuch’s (1996) influential work “A Woman Is Not a Man”*: The Culture of Gender and Generation in Soviet Russia* illustrates that, although revolutionary works promising gender equality were being published, early Soviet structures such as the *Komsomol* youth organization reproduced a deeply masculinized political culture. Participation of men in family, social, and political issues was framed as rational, disciplined, and ideologically mature, while participation of women was linked to backwardness or emotional excess.

Ashwin’s (2000), *Gender, State, and Society in Soviet and post-Soviet Russia* perceives that Russian masculinity and political institutions are two inseparable entities used to legitimize authority. She argues that men’s social value is entirely based on their ability to endure hardship, labor, and systemic instability, rather than on their emotional expressiveness. Aswhin argues that restraint is normalized as moral strength over repression. This argument provides a framework to study Rostov’s composure as an absence of emotion. Thus, masculinity is measured through survival rather than success in the Tsarist era.

On the other hand, Oushakine (2009), with his critical essay, “The Patriotism of Despair,” introduces the idea of a post-Soviet affective condition. This condition is marked by loss and nostalgia as Count often reminisces about his wounded national identity. Oushakine depicts that men were encouraged to perform stoicism even in the face of social collapse. Their emotional articulation of feelings was redirected toward the cumulation of repressed mourning. Oushakine further directs his attention to “affect,” relying on masculinity not as a role or ideology but as a restraint on both survival and moral expectation. This framework helps clarify the collective suppression of emotion among Russian men, leading to the understanding that emotional openness is seen as destabilizing masculine culture.

Substantiating Oushakine, Janey et al. (2009), who studied Ukrainian masculinities, identify similar archetypes such as the *stoic protector* and the *reserved man*. This masculinity comes with a cost of endurance, dignity, and emotional silence—the traits that are historically valorized across all Slavic cultures. Thus, affection is expressed through protection rather than tenderness. In addition to these scholars, some religious works contribute to the concepts of emotional restraint. Zorgdrager’s (2013) “Homosexuality and hypermasculinity in the public discourse of the Russian Orthodox Church: an affect theoretical approach” argues how post-Soviet Orthodox Church narratives have constantly encouraged a patriarchal version of masculinity which is grounded fervently in authority, heterosexuality, and moral guardianship. Under such an ideological backdrop, the dominant form of masculinity became a moral performativity of control, resonating with Connell’s hegemonic model.

Russian society, like any other imperial and colonial culture, has produced masculinity through systems of discipline and ritual. The concept of “gentleman” became one such institutionally manufactured ideal within this tradition, often dignified, self-contained, courteous, authoritative, yet emotionally unreachable, being both the symbol and victim of the hegemonic archetype. Huggins (2012), in his work *Manufactured Masculinity*, provides “a strong reminder that masculinity has always been constructed and manufactured within specific institutional settings. Masculinity is not a mere biological identity since cultural and social power structures, while usually diverse, play a key role in shaping it” (Huggins, p. 150). Thus, the reviewed scholarly works establish the theoretical conditions for understanding and analyzing the masculine transformation, yet they leave a significant analytical gap at the intersection of these frameworks. This study addresses the research gap by asking what cinematic and relational mechanisms contribute to the transformation of hegemonic masculinity into an ethical masculinity, grounded in care?

## 3 Methods

The study employs a qualitative methodology combining critical masculinity studies with audiovisual textual analysis. The primary source for the study is Sam Miller’s eight-episode television adaptation of *A Gentleman in Moscow*, produced by Paramount+. It focuses on the critical analysis of all eight episodes in full, giving close attention to recurring patterns in visual composition, character interaction, and dialogue. The serial form of this text plays a methodologically significant role, as Mittell (2015) points out that the character transformation in complex television narrative accumulates meaning differently from that in a standalone film, because it does not focus on a single pivotal scene. Giving attention to how meaning develops cumulatively, the study examines Rosov’s masculine transformation across the arc of eight episodes. As for the visual analysis, the study relies on Bordwell and Thompson’s (2010) framework of mise-en-scène and is followed by Kozloff’s (2000) dialogue analysis, which insists that visual context and spoken language go hand in hand to derive a meaningful interpretation rather than examining them in isolation. This study examines the performance of masculinity through the spaces it occupies, the sentiments it portrays, and the connections it forms among characters. The study draws on Connell’s notion of hegemonic masculinity, Bourdieu’s idea of habitus, and recent ideas about caring and affective masculinity from Hanlon and Elliot. By closely studying details, such as gestures, camera angles, tones, and the script, the study incorporates Denzin and Lincoln’s (2018) analytical procedure to converge on the visual, performative, and dialogic patterns. This helps the study to follow how masculinity changes throughout the series.

## 4 Findings and analysis

### 4.1 Hegemonic masculinity in the form of aristocratic composure

As the series begins, the Revolution of 1917 leaves Russia in a state of political upheaval, leading to the execution of the Royal families. The shift of power from the Tsarist aristocracy to the Leninists pushes Count Alexander Rostov to abandon his mansion. Although the beginning proceeds with the downfall of the aristocrats, Rostov personifies a historically specific form of masculine ideal called hegemonic masculinity, one that is built upon aristocratic composure, emotional restraint, and a sense of moral superiority over the weaker ones, rather than relying on violence, dominance, and physical aggression. Despite being nervous at the beginning of the first episode, glancing from a corner at his fellow aristocrats getting sentenced to death, he maintains a calm demeanor in front of the judges and the panel members. He asserts his authority through his poise, silence, and self-control. The opening tribunal scene of the very first episode establishes Rostov’s hegemonic masculine identity through both the visual composition and verbal register, which Kozloff (2000) identifies as the dual register of audiovisual meaning. As the trail begins, one of the members enquires about Rostov’s occupation. The framing of the camera places him at the center of the composition despite being an accused. The overall lighting remains cool and controlled, along with his witty remark, “it is not the business of gentlemen to have occupations” (*A Gentleman in Moscow*, Episode 1, 02:30), which reveals a masculinity that derives authority not from labor or achievement but from social distinction. This aligns with Connell’s (2005) account of hegemonic masculinity that is not monolithic but culturally contingent, tailoring itself to the dominant class logic of any society.

In the early scenes, Rostov’s masculinity aligns with Bourdieu’s (2001) concept of *habitus*: the internalization of gendered and classed expectations that is reflected in posture, tone, and restraint. As he comes to know that the poem, which apparently saves his life, was written by his close companion, Mikhail Fyodorovich Mindich, aka Mishka, in the face of the jurisdiction, his controlled gestures, measured speech, and refusal to display any sort of panic or surprise reveal a masculinity trained to contain emotional exposure, which is considered undignified. Specifically, this containment departs from neutrality and serves as a marker of superiority. Thus, the emphasis on bodily disposition rather than insisting on the containment as a conscious strategy is analytically precise to Bourdieu’s (2001) concept of *habitus*. The placidity presented here is not a mask that covers authentic feelings but a genuine product of *habitus*. According to Bourdieu, Rostov does not need to proclaim power overtly; his placidity itself commands respect.

Hearn (2014) emphasizes the strain created by material-discursive forces, a masculine identity produced through the simultaneous interaction of physical environments and social discourse. According to his work, what is enacted bodily and what is said about it work together to produce masculine meaning. The grandeur of the Metropol hotel’s public space, the formality of its dining rituals, and the deference of the staff intensify the material conditions that provide the discursive stage. After the sentencing, Rostov loses access to all his wealth and assets; he is stripped of material privilege. This implies that removing the strain on society might pave the way for the transformation of the masculine ideal. Hitherto, his composure allows him to retain symbolic authority, which implicates the multitude of masculinities. In early episodes, he maintains a masculinity that remains hierarchical: his observation of others from a distance without engaging them; his resemblance of generous judgement rather than intimacy when he offers which wine would be best fit with which food to another diner in episode 3; and his polite but consistent distancing from emotional entanglement with others and especially Nina Kulilova in the beginning of their bonding, echoes a masculine disposition which aligns with Ashwin’s (2000) description of Russian masculine ideals across both Tsarist and Soviet contexts. Ashwin further argues that these masculine ideals have consistently valorized dignity and endurance over emotional expressiveness, thereby narrowing masculine worth to the capability to endure hardship.

However, the series smoothly exposes the limitation of this aristocratic composure. Initially, Rostov’s restraint operates as emotional insulation, but it gradually evolves into ethical involvement. His refusal to fully engage with others’ vulnerabilities, especially his initial halfhearted help for the maid’s child, reveals how aristocratic masculinity still aligns with hegemonic distance. Although he helps others benevolently within the hotel, he is emotionally detached. His kind and calm demeanor with the rude waiter and the soldiers who mistreated him reflects his gentle mannerism, which is yet to be relational in care. Masculinity presented by Rostov here is moralized but not ethical.

Visually, the series reinforces the hegemonic masculinity through symmetrical framing and controlled pacing of Rostov. As Laura Mulvey’s (1975) theory of the male gaze suggests, Rostov’s character is centered throughout the series. He stands as the perspective that aligns spectatorship with heterosexual male desire. In the initial stage of the series, he is often isolated within the frame, separating himself from others while showcasing mastery over himself. Rostov becomes the active bearer of the gaze, driving the narrative and controlling its direction. Despite the female characters being positioned as passive spectators in the beginning, Miller changes this stance by the latter episodes. Thus, his early appearance bearing hegemonic composure provides the raw material from which an alternative masculinity will later emerge. This transition that follows is the pivotal one from control to responsibility and distance to care, changing Rostov’s character.

### 4.2 Confinement as masculine deconstruction

The confinement of Rostov in the hotel’s attic operates as a systematic dismantling of hegemonic authority. In episode 1, following the conversation with his friend Prince Nikolai at the bar, Count reminisces about his parents’ funeral. While doing so, he visualizes the coffins being nailed firmly. This reflects his current scenario of how he’s nailed down within the hotel and metaphorically symbolizes the death of hegemonic masculinity. The aristocrat who is once accustomed to commanding social space is visually brought down within a tiny enclosure as the coffin. This spatial contraction embodies what Connell (2005) identifies as the fragility of hegemonic masculinity that occurs when the institutional foundation is withdrawn. Masculinity does not exist autonomously in this framework. It depends upon recognition, privilege, and structural reinforcement, and when these conditions vanish, masculinity enters a state of exposure.

Concurrently, the stripping away of power is evidently happening to the majority of the people that Count knows of, beginning with his friend Prince Nikolai. The fragility of this aristocratic masculine identity is visually and verbally portrayed during the brief exchange between the Count and Nikolai in the hotel lobby. Keeping close alternating shots that isolate each from each other, rather than uniting them in a shared frame, is a visual choice that conveys the dissolution of the social world that once connected them. Nikolai’s face becomes frightened, and his response to Count’s inquiry about his palace mirrors Rostov’s state of being.

Petrov: Alexander Ilyich? Is it really you?
Rostov: Prince Nikolai, my dear friend, it’s so wonderful to… [Interrupted by Petrov].
Petrov: It’s just Nikolai now (*A Gentleman in Moscow*, Episode 1, 17:34–17:44).

At the narrative level, this scene implies the loss of power as the price of becoming part of a moving troupe. He lost not only his title but also his identity as a royal ideal authority, just like Count Rostov did. Although many, once powerful men around Count seem to crumble and fall from their thrones, he holds his stature without breaking down. However, it drives him to a tension that eventually leads him to his transformation. The mise-en-scène of the confinement in the attic articulates this exposure through bodily restriction. Rostov’s upright posture and controlled gestures persist, yet they circulate without effect. His habitual act of straightening his cuffs and meticulous arrangement of objects within his room resemble what Bourdieu (2001) would address as embodied residues of class distinction. Forms of habitus that remain functioning even when their social efficacy has disintegrated. These gestures of Rostov no longer produce authority rather, they appear as echoes of a vanished hierarchy. Thus, masculinity becomes a memory inscribed on the body instead of a living social force.

Gender identity requires continual reiteration within a supportive symbolic environment. Butler’s (2004) concept of performativity illustrates “that through the practice of gender performativity, we not only see how the norms that govern reality are cited but grasp one of the mechanisms by which reality is reproduced and altered in the course of that reproduction” (Butler, p.218). At this point, masculinity is understood as a citational practice that depends upon a supportive symbolic environment. It needs an audience, institutional reinforcement, and the material conditions that make each repetition legible as masculine authority. In Rostov’s case, the confinement removes the audience necessary for masculine performance and reinforces institutional and material conditions with resignification. Although his gestures repeat as a habit, they fail to signify the masculine authority. Thus, the Count does not immediately abandon aristocratic discipline, he confronts its emptiness little by little. The visual representation of all rooms, along with the lobby and diner, in a bright colorful manner, while showing his room alone in a dark teal shade, resonates the emptiness and pitiful value of hegemonic masculinity when its power is stripped off.

The dinner scene in the middle of episode 1 intensifies this emptiness of hegemony, where Rostov is seated alone at a table in the hotel dining room. The camera angle in the initial framing, placing him in a wide shot, emphasizes the shallowness of his surroundings Although being treated slightly rudely by the waiter, he insists on ceremonial etiquette despite his reduced circumstances. As Nina arrives, the tone gets warmer, and the spatial isolation is momentarily relieved by the presence of another person. Rather than expressing in dialogue, his profound gesture of unfolding his napkin with utmost care and sharing his food with Nina, as although it is presiding over abundance, perfectly aligns with what Bordwell and Thompson (2010) describe as the expressive function of performance within the mise-en-scène. This scene dramatizes what Yusupova (2017) describes an Russian masculinity “revolutionized from above” (p.11), in which political transformation forcibly reorganizes masculine identity. The significance of Yusupova’s framework here highlights the temporal lag between political transformation and embodied masculine transformation. Revolution, in the usual sense, can abolish titles and confiscate estates overnight, but the immediate dissolution of the bodily disposition seems far-fetched. To substantiate the argument, Ashwin’s (2000) idea of Soviet masculinity as a citizenship of endurance highlights further insight into it. Russian masculinity is defined by endurance in the face of hardship. Rostov’s constant determination to uphold his decorum mirrors the Russian cultural logic of restraint, as mentioned by Aswhin. The civility he displays creates a tension between inherited dignity and present powerlessness.

*The Patriotism of Despair* by Oushakine (2009) frames this scenario as affective displacement, a post-imperial emotional condition in which the grief for a lost social order is redirected into nostalgia and attachment to the cultural practices of the vanished world. This framework sheds light on episode 1, as Rostov narrates how a duel between two takes place in front of Nina. While doing so, he recalls the duel that took place before the revolution, which resembles his attachment to lost order. This reflects reliance on nostalgia portrayed by post-imperial masculinity when its structural power erodes. His refined gestures and frequent reminiscence of events embody a longing for coherence over resistance. The series does not ridicule this attachment. Miller makes the camera linger on Rostov’s movements with tenderness, granting his struggle emotional legitimacy. Thus, presenting masculinity as a wounded continuity.

The cinematic compassion showcased here prevents Rostov’s confinement from becoming a mere act of discipline. It turns spatial restriction into an act of ethical exposure that lets Rostov break free from hegemony. He is forced to confront a masculinity that is stripped of its privileges and advantages. Masculinity gets pushed into a reflective phase by dissolving the very link between authority and entitlement. In this phase, “power” is neither exercised nor reclaimed, creating a space for deliberation. This paves the way for ethical and relational engagement to replace structural dominance. The transition is marked as the first step toward ethical and affective masculinity.

### 4.3 From stoic control to affective masculinity

As the confinement stands as the initial stage of the collapse, Nina’s relationship paves the way for Rostov’s transformation from inherited authority. It institutes the re-education of emotion. The attic room strips him of his authoritarian power, while Nina’s presence confronts him with emotional immediacy. From the beginning of the series, Nina’s surprising visits and her approach to Rostov let him express his true self. Masculinity here begins its inward turn. This corresponds closely with Levant and Wong’s (2017) concept of *normative male alexithymia*, which holds that emotional suppression is at the core of hegemonic socialization. Men are taught to suppress their feelings, which is equated with maturity and strength. Rostov initially embodies this logic by initiating regular chores with himself that eventually drive him to emotional insulation.

The solitude he produces is not incidental but a symptom of the inevitable consequence of a masculinity that has solely learned to organize experience around control rather than connection. However, Butler (1993) indicates that the disruption of the existing masculine authority and the solitude Rostov undergoes do not simply mark an ending but an opening. When the citational environment, such as the aristocratic social world, has left him no role to perform, the compulsive repetition of his daily rituals begins to lose its performative force. This interruption of the citational chain is precisely what creates an opening for resignification. His loneliness does not last long as he starts to open up with his emotional space, particularly with Nina’s daughter in the latter end of the series, where the majority of his daily chores will be accompanied by Sofia. His gradual engagement with Nina and latter role of a father to Sofia introduces new citational conditions under which habitual gestures are resignified.

The quick interactions that he has with Nina begin as an instructional performance. To satisfy her curiosity, he teaches etiquette, manners, and restraint, reproducing the same disciplinary masculinity he inherited. Rostov slowly becomes an apprentice to Nina’s emotional openness rather than her becoming his pupil. The early conversations between them in the series are framed in mid-shots that reduce hierarchical distance. He begins to listen more than to speak, and his posture shifts forward, his gaze softening. The physical transitions portrayed here directly resemble Hanlon’s (2022) affective equality. A relational condition in which emotional hierarchy shifts toward reciprocal engagement, indicating that one does not consistently hold the position of emotional authority. The change in Rostov’s posture, shifting forward, and the softening of his gaze precisely mark this condition from superiority to reciprocity.

The series slowly unfolds several incidents, such as repetition of shared meals, quiet conversations, and moments of curiosity with Nina. These subtle symbolic elements help the narrative resist sudden shifts and favor gradual transitions. Levant and Wong (2017) differentiates between emotional suppression and emotional regulation in his work, *The Psychology of Men and Masculinities*, where he identifies that suppression leads to blockage of emotional experience from reaching its conscious awareness entirely, while regulation helps to experience emotions to their full potential by modulating their expression appropriately to context and relationship. What Rostov undergoes is an emotional regulation that allows his feelings to register internally. He does not abandon his restrained nature. He learns to internalize it differently. This paves the way for his journey toward emotional responsiveness. Furthermore, in episode 2, Nina reveals that she possesses a secret master key. The key that opens any door within the hotel. Meanwhile, Nina metaphorically serves as the key that opens Rotov’s empathetic nature. The affective masculine education is once again enforced through cinematic language. Although the early scenes from the series emphasize spatial isolation and rigid framing. Miller gradually replaces wide compositions with intimate medium shots. As the camera moves closer to Rostov’s emotional orientation, it slowly erodes his authoritative power. The lighting shifts from cold blues to warmer tones, visually representing the emotional shift from cold-heartedness to the warmth of emotion.

This evolution matures to a critical point toward the end of the series in Episode 8. Rostov tears up openly, the moment when Anna departs from him in the hope that he will catch the train. Rendered with minimal dialogue and sustained close-up during their final dance in the hotel, he allows his emotional expression to surface in the visual center of the scene without restraining it. Rostov’s grief does not diminish his authority; it exemplifies Butler’s (1993) theory of resignification, which suggests that although historically “tears” are coded as weakness within hegemonic masculinity. She also states that resignification does not ignore or eradicate history but rewrites it in a new context. It acquires different meanings and offers a powerful lens. Rostov confirms his ethical capacity by making emotional vulnerability his moral strength toward the end. Rostov’s tears are not a deviation from his masculine identity, nor does it diminish his authority. As it emerges from years of relational and emotional transformation established across the preceding episodes, it resignifies emotional vulnerability as moral strength for him.

As Hanlon (2022) advocates, a man’s affective competence emerges when he learns to hold his emotions without dominating or withdrawing from them. This integration marks the transition of Rostov’s character from stoic control to affective masculinity. He begins to experience emotion as responsibility rather than a threat or weakness. Thus, Masculinity is not replaced but re-signified. The education of feeling with emotional openness prepares the ground for subsequent transformation in Rostov’s romantic intimacy and paternal care. Emotional restraint has shifted in meaning and now serves as the basis for how relational masculinity is constituted.

### 4.4 Love and the gentle undoing of pride

The relational transformation that takes place within Rostov gradually occurs throughout the eight episodes as a continuum. The relationships that he has with Anna Urbanova as a partner and with Sofia as a father do not simply soften his masculinity. They stand as the pivotal point that reconfigures his moral orientation. Both relationships play their part in detaching hierarchical intimacy and reorganizing authority around. Rostov’s relationship with Anna marks the first decisive rupture in his aristocratic emotional economy. During their early encounters, Rostov approaches Anna with aristocratic distinction. His behaviors, such as politeness, wit, and composure, are marked by subtle superiority. Toward the end of episode 2, right after their first physical contact, as they keep questioning each other, Anna’s tone is measured but pointed to expose the hierarchical assumptions embedded in Rostov’s apparent warmth:

Rostov: How could I possibly relinquish the memory of this?
Anna: hmm…. Or of all the princesses and duchesses, who have come before me?
Rostov: Oh. Some, not all.
Anna: I’m surprised you countenance sleeping with a lowly actress.
Rostov: Well, as everyone keeps telling me, times have changed.
Anna: What exactly do you mean by that?
Rostov: Very little. (*A Gentleman in Moscow,* 32:03–32:32)

Though Rostov’s confinement destabilizes his public authority, masculinity keeps reproducing hierarchy through his private habits that are exposed during his intimacy. To Anna’s remark, his reply of “times have changed” enforces his hegemonic assumption that, being a nobleman confined with limited sources, he needs to adjust to what he is given, as if Anna’s presence is a concession to circumstance. He refuses to recognize her as an equal, and the camera’s close two-shot framing adds to it by creating an ironic counterpoint. This reflects what Bourdieu (2001) describes as distinction through distance. His witty comment reveals the hegemonic nature deeply suppressed within him, which is soon confronted and corrected by Anna’s actions. For Rostov, until he truly realizes that he’s in love with Anna, he admires her but does not see her as his equal.

Unlike others, Anna’s confrontation seems to question the hegemonic masculinity that sees women as sexual objects. Her question of “because I would be beneath a man like you?”(*A Gentleman in Moscow, episode 2,* 32:33) disrupts the posture of the Count’s dominant and authoritative superiority. During this confrontation of hierarchy, the camera angle cuts to a close-up shot of Anna alone, giving her face the same sustained visual attention that was previously reserved for Rostov. She expresses her desire and affection along with frustration, without deflection, inhabiting vulnerability as agency rather than weakness. Her femininity, which is pragmatic and self-possessed, forces Rostov to confront the limitations of his emotional capacity governed solely by etiquette, and, at the narrative level, the shift takes place from romantic interest to ethical interlocutor. As Hanlon’s (2022) relational labor of affective equality suggests, the action of refusing to absorb another person’s hierarchical assumptions in silence initiates them to engage in reciprocal acknowledgment. Thus, Rostov learns that he cannot sustain intimacy through composure without reciprocity. Gradually, as he keeps trying to contact her after being served with many cold shoulders, his guarded restraint gives way to relational presence.

Rostov’s voice softens, and his gaze lingers along with his gestures loosening stiffness. It reveals the gentle shift through bodily recalibration. Politeness is redirected from superiority to attentiveness. In the midsection of episode 3, after trying to re-establish contact with Anna several times through passing notes with the help of hotel staff and oblique signals, Rostov finally decides to approach her directly. However, to his surprise, he learns that she waited for his direct approach over all the distant signals that he attempted. The camera frames him with medium shots, emphasizing the tension the series has built around Rostov’s inability to be present without mediation. At the visual level, Anna’s sustained gaze into the camera reflects her resistance to the acts influenced by hegemony that sees women as a sexual commodity. Her statement, “You didn’t send yourself” (*A Gentleman in Moscow,* episode 3, 22:04), undermines Rostov’s entire authoritative and dominant mindset. This relational shift in Rostov exemplifies Hanlon’s (2022) concept of affective equality, where emotions are shared through moral labor. As Rostov reunites with Anna and hears about her father’s sacrificial love, he learns to inhabit vulnerability without relinquishing dignity. Slowly, his love toward Anna changes from possession to presence. He relinquishes the final residues of his aristocratic pride through Anna, preparing the ethical ground for a more demanding transformation.

The final stage of the transformation occurs through Rostov’s guardianship of Sofia. Her presence in the latter episodes reinstigates masculinity as a sustained caregiving responsibility. Rostov’s role as a father is neither biological nor ideological. Rostov’s masculinity is no longer tested through emotional openness alone but through ethical enactment and long-term commitment. Rostov’s interactions with Sofia throughout the series are marked by attentiveness rather than basic instructions. The ethical maturity of Rostov’s transformed masculinity finds its clearest expression during the beginning of the fifth episode as Rostov and Sofia have their usual breakfast in the secret room. When he notices the doll that Sofia carries and enquires the name of it, she replies implying that it has no name. The quiet marker of how far Rostov has traveled from the emotional inaccessibility to a man who is engaged in careful, attentive conversation is profoundly visualized through his teaching about the importance of having a name and how to address someone in a conversation during their absence. Through close two-shot framing with genuine spatial equality, neither Rostov nor Sofia elevated above the other. Thus, this gentle guidance and careful listening assures Rostov’s paternal orientation countering his initial aristocratic instructions.

Elliott’s (2016) formulation of ‘caring masculinity’ should not be misread as a surrender or abandonment of masculine strength but as its fundamental reworking. A movement that shifts away from the dominant and hegemonic form of masculinity toward the positive emotional expressiveness, interdependence, and relational attentiveness. The discipline, attentiveness, and composure that once assisted Rostov’s hierarchical distance are now entirely directed toward caring for the wellbeing of Sofia. Although the initial stage of the narration opens up with trial by isolation, it is caregiving that constitutes its ethical resolution in him. The relational masculinity that replaced hegemonic masculinity reaches its fullest ethical articulation only when care demands risk. The final episode depicts Rostov’s strategy for Sofia, Anna, and himself to escape and leave the Soviet Union. However, he quietly rigs a plan to ensure that Sofia and Anna will have the easier route out. The emotional maturity gets translated into moral courage through his selfless act. Rostov’s decision is neither an impulsive act of resistance nor a wistful rebellion. However, a well-measured and morally grounded commitment is shaped by years of practicing relational responsibility.

## 5 Conclusion

The study concludes that *A Gentleman in Moscow* (Miller, 2024) stages masculinity in a new light through gradual ethical transformation. It argues that the series defies the traditional dominant side of masculinity while retaining masculine ideals, such as discipline, composure, and responsibility. Miller’s cinematic grammar cautiously makes the idea of hegemonic masculinity drift away and brings caring masculinity into reality. The detachment that Rostov feels in the early episodes reflects the loss of power and authority. While the latter episodes reveal the empathetic shift in his character. This helps him to reciprocate feelings, disregard romantic hierarchy, and care for others. Thus, it provides a blueprint for modern men to change their idea of moral and emotional behavior while retaining their discipline and composure.

This series situates Rostov’s transformations within the context of Russian culture, which characterizes strength, loss, and the suppression of emotions as masculine ideals. Rostov’s ability to restrain himself reflects the stoic and the self-reliant traditions noticed in studies about Russian men. Masculinity is thus not rejected or idealized but reworked by practicing what’s right. It also proposes a model of masculine maturity rooted in responsibility over control. The study finally facilitates an understanding of masculinity that examines ethical masculinity as a unique entity. Sam Miller’s adaptation of *A Gentleman in Moscow* (Miller, 2024) thus highlights the changing masculinity. Being a man demands continuous adaptation across relationships, responsibilities, and time.

The upcoming research studies can use this framework in comparative studies focusing on confinement narratives or aging masculinities. It can also be used in many historical and cultural ways to show how manhood shows up when history makes things tough. Since the contemporary media prioritizes sentiment and compassion, this perspective argues on how manhood is represented and lived out with others. Thus, *A Gentleman in Moscow* (Miller, 2024) provides a calm yet strong view of what manhood ideal can be.

### 5.1 Limitation of the study

This study faces several limitations. Starting with the analysis section, which relies completely on a single visual text with sets of theories. Even though the narrative is rich and the representation remains aesthetic, not all contemporary perspectives of men can draw a generalized conclusion. The other limitation is the adaptation’s setting. Given its setting in the historical turmoil of post-revolutionary Russia, contemporary viewers may interpret it differently depending on their cultural background, gender position, or ideological perspective.

## Data Availability

The original contributions presented in the study are included in the article/supplementary material, further inquiries can be directed to the corresponding author.
